# Assessing and Stabilizing Aberrant Neuroplasticity in Autism Spectrum Disorder: The Potential Role of Transcranial Magnetic Stimulation

**DOI:** 10.3389/fpsyt.2015.00124

**Published:** 2015-09-09

**Authors:** Pushpal Desarkar, Tarek K. Rajji, Stephanie H. Ameis, Zafiris Jeff Daskalakis

**Affiliations:** ^1^Department of Psychiatry, Centre for Addiction and Mental Health, University of Toronto, Toronto, ON, Canada; ^2^Temerty Centre for Therapeutic Brain Intervention, Centre for Addiction and Mental Health, Toronto, ON, Canada; ^3^Department of Psychiatry, The Hospital for Sick Children, University of Toronto, Toronto, ON, Canada; ^4^Research Imaging Centre, Campbell Family Mental Health Research Institute, The Centre for Addiction and Mental Health (CAMH), Toronto, ON, Canada

**Keywords:** autism spectrum disorder, transcranial magnetic stimulation, neuroplasticity, EEG, treatment

## Abstract

Exciting developments have taken place in the neuroscience research in autism spectrum disorder (ASD), and results from these studies indicate that brain in ASD is associated with aberrant neuroplasticity. Transcranial magnetic stimulation (TMS) has rapidly evolved to become a widely used, safe, and non-invasive neuroscientific tool to investigate a variety of neurophysiological processes, including neuroplasticity. The diagnostic and therapeutic potential of TMS in ASD is beginning to be realized. In this article, we briefly reviewed evidence of aberrant neuroplasticity in ASD, suggested future directions in assessing neuroplasticity using repetitive TMS (rTMS), and discussed the potential of rTMS in rectifying aberrant neuroplasticity in ASD.

## Introduction

Autism spectrum disorder (ASD) is a complex neurodevelopmental disorder characterized by persistent deficits in social communication and interaction and stereotyped behaviors, interests, and activities [*Diagnostic and Statistical Manual of Mental Disorders*, 5th Edition (DSM-5)] (1). The most recent US Centers for Disease Control and Prevention data estimate that ASD now affects 1 in 68 children (2). These data establish ASD as the most common neurodevelopmental disorder. Thus, the social, clinical, and economic burden of ASD is tremendous.

Since the turn of the century, significant advancements have been made in ASD research, and a range of macro- and micro-structural, neurochemical, functional, anatomic, and genetic abnormalities have been proposed [see reviews by Rubenstein and Merzenich (3), Parellada et al. (4), Chen et al. (5), Ameis and Catani (6)]; however, despite gaining important leads, the exact etiology of ASD is still unknown and successful treatment remains elusive. Thus, there is an urgent need to explore novel and effective investigational and mechanism-driven treatment paradigms for ASD.

One mechanism that has recently received a large amount of support suggesting its role in the pathophysiology of ASD is aberrant neuroplasticity (7, 8) In fact, several lines of evidence from genetic (9–13) to animal model (7, 14), neuroimaging (15, 16), and brain stimulation (17, 18) research have all begun to implicate aberrant neuroplasticity in ASD. One neuroscientific tool that has become a widely used, safe, and non-invasive way to probe aberrant neuroplasticity is transcranial magnetic stimulation (TMS) and repetitive TMS (rTMS). Perhaps a fair example of this is the use of TMS/rTMS in the study of Parkinson’s disease [see review by Shukla and Vaillancourt (19)], depression (20), and schizophrenia (21). The diagnostic and therapeutic potential of rTMS in ASD is beginning to be realized. In this article, we will briefly review evidence of aberrant neuroplasticity in ASD, suggest future directions in assessing neuroplasticity using rTMS, and discuss the potential of rTMS in rectifying aberrant neuroplasticity in ASD.

## Aberrant Neuroplasticity in ASD

Before describing the evidence in favor of aberrant neuroplasticity in ASD, it may be worthwhile briefly revisiting neuroplasticity first. Neuroplasticity refers to neuron’s ability to reorganize and alter their anatomical and functional connectivity in response to the environmental input. Long-term potentiation (LTP), which involves a net increase in synaptic efficacy, and long-term depression (LTD), which indicates a net decrease in synaptic efficacy, are the two prototypes of neuroplasticity (22).

In a simplistic model, LTP is mediated by glutamate via *N*-methyl-d-aspartate (NMDA) receptors (23). The basic process of LTP generation involves the removal of the Mg^2+^ block of the post-synaptic NMDA receptors by a strong wave of depolarization in the dendritic spine, leading to a rapid inflow of Ca^2+^ that activates several kinases, eventually leading to the generation of LTP. Similarly, LTD too perhaps is dependent on NMDA receptors. The mechanism of LTD generation, however, requires milder activation of post-synaptic NMDA receptors, which leads to an intermediate intracellular Ca^2+^ elevation (23). One key regulator of LTP and LTD is gamma-aminobutyric acid (GABA) released by the inhibitory interneurons (24). At the synaptic level, the fine balance between excitation (mediated by glutamate) and inhibition (mediated by GABA) could be crucial for optimal level of neuroplasticity (25).

### Evidence from the structural neuroimaging studies in ASD

Most of the symptoms of ASD develop in the first few years of life when synaptic development and maturation are occurring at a rapid rate, and one of the most consistent morphological findings that emerged from the structural neuroimaging studies in ASD is early brain overgrowth (15) [also see review by Courchesne et al. (16)]. Such atypical brain enlargement appears to be most pronounced between 2 and 5 years of age (16), and it preferentially affects the frontal and temporal cortices (5). Furthermore, recent evidence indicates that atypical cortical development in ASD subjects persists beyond toddlerhood. In particular, evidence of cortical thinning has been observed among adolescents and young adults (26). These observations led to the hypothesis that ASD is associated with a significant disruption of the typical synaptic maturation and plasticity (5).

### Evidence from the genetic studies in ASD

Of all the proposed neurobiological theories of ASD, the potential contribution of genetic factors is backed by a large body of evidence [see review by Chen et al. (5)]. It is important to note that many ASD-associated genes reported by genome-wide association studies encode proteins related to synaptic formation, transmission, and neuroplasticity, and results from recent genetic studies involving ASD clients have consistently linked mutations involving several genes supporting synaptic maturation and neuroplasticity. The examples of such mutations involve genes critically involved in (a) synaptic maturation, e.g., neuroligin 3 and 4 (10), c3orf58, NHE9, and PCDH10 (13); (b) neuronal migration, e.g., CNTNAP2 (12); and (c) dendritic development, e.g., SHANK3 (12).

### Evidence from animal models of ASD

Further evidence of aberrant neuroplasticity in ASD is shown by animal models. Perhaps one of the best known among these models is the valproic acid (VPA) rat model of autism. This model predicts that brain in ASD is likely to be hyperplastic. It has been found that, following a Hebbian Pairing Stimulation protocol, the amount of post-synaptic LTP measured in the neocortex and the amygdala doubled in VPA-treated rats compared with controls (14). However, other animal models utilizing genetically modified mice showed that ASD brain could be characterized by both impairment and enhancement of neuroplasticity. For example, Shank3(G/G) mice (27) and mice with MECP2 mutations (model of Rett’s syndrome) (28) were shown to have cellular hypoplasticity, but mice with neuroligin-3 mutation were associated with hyperplasticity (29). Such divergent outcomes with regard to the direction of neuroplasticity in these animal experiments could be due to the nature of the genetic modifications used and their impact on the brain substrates of neuroplasticity. Nevertheless, a key insight emerging from these animal models is that if the brain becomes too much or too less plastic (i.e., hyper or hypo), cognition and behavior will be affected. It has been suggested that an optimum level of plasticity is necessary for optimal performance (30), and this process essentially involves keeping excitability within a normal physiological range (31).

### Excitation/inhibition imbalance in ASD

Perhaps one of the widely cited neurobiological models in ASD over the past decade is the increased excitation/inhibition ratio in ASD brain (3). It has been suggested that the excitation–inhibition imbalance could be the key determinant of neuroplasticity abnormalities in neurodevelopmental disorders such as ASD (32), and a deficit in the inhibitory neurotransmission has been implicated in the etiopathogenesis of ASD [see review by Baroncelli et al. (25)]. It is believed that such deficits could develop during neuronal maturation (25). At the synaptic level, abnormally increased NMDA-mediated state of excitation, and/or abnormally reduced GABA-mediated inhibition, may lead to abnormally increased neuronal excitability and neuroplasticity. In fact, studies involving subjects with ASD have shown that excitatory glutamate receptors (NMDA and metabotropic glutamate receptor 5) are overexpressed, whereas inhibitory gamma aminobutyric acid A (GABA_A_) and B (GABA_B_) receptors are underexpressed in the ASD brain (25, 33). Additionally, post-mortem studies of minicolumnar morphometry in subjects with ASD also demonstrate a significant reduction of the peripheral neuropil space, which is the site of GABA-ergic lateral inhibition in the brain (34).

Transcranial magnetic stimulation has also been used to investigate excitation–inhibition imbalance in ASD. Specifically, paired-pulse TMS paradigms, involving the “pairing” of a “conditioning stimulus” with a “test stimulus” at different interstimulus intervals, have been used to assess cortical inhibition (CI) and facilitation. CI is the neurophysiological process in which inhibitory GABA-ergic interneurons selectively attenuate the activity of pyramidal neurons in the cortex. It has been suggested that CI is key to the regulation of neuroplasticity, and the therapeutic effects of rTMS could be mediated by the induction of local changes in CI (35). Emerging evidence indicates that post-synaptic GABA_B_ receptor-mediated CI is crucial for the regulation of neuroplasticity. GABA_B_ regulates neuroplasticity in two ways: (a) they contribute to the regulation of inhibition by mediating long-lasting inhibitory post-synaptic potentials (IPSPs) and (b) they reduce GABA_A_ receptor-mediated inhibition through presynaptic autoinhibition of inhibitory interneurons (36). Using paired-pulse TMS paradigms, studies have found evidence for excitation–inhibition imbalance in a subgroup of individuals with ASD (37, 38). Other studies have shown no abnormality in CI (18, 39) or a heterogeneous response to this paradigm (40). The heterogeneity in these findings reflects the known heterogeneity of ASD at both the behavioral and the physiological level.

## rTMS in the Assessment of Neuroplasticity in ASD

Repetitive TMS, which involves repetitive delivery of pulses (>1 Hz), is used to modulate cortical activity for investigative and therapeutic purposes [see review by Kobayashi and Pascual-Leone (41)]. rTMS has been increasingly used to study neuroplasticity in humans. The basic premise is that rTMS can modulate activity in the targeted brain region for a duration that can outlast the effects of stimulation itself (30). It is believed that rTMS induces such lasting changes in the brain through altering neuroplasticity mechanisms (42). So far, two rTMS paradigms – theta-burst stimulation (TBS) (17) and paired associative stimulation (PAS) (18) – have been used to assess neuroplasticity in ASD.

### Theta-burst stimulation

Theta-burst stimulation involves the delivery of a burst of three pulses at 50 Hz (i.e., 20 ms between stimulus) repeated at intervals of 200 ms (i.e., 5 Hz, hence called theta-burst) (43). TBS comprises two well-established patterned stimulation protocols – continuous TBS (also known as cTBS) and intermittent TBS or iTBS. cTBS paradigm involves the delivery of continuous uninterrupted TBS for 40 s. In the iTBS paradigm, a 2-s train of TBS is repeated every 10 s for a total of 190 s. However, the total number of pulses delivered may vary from one study to another. In the original study, Huang et al. (43) used 600 pulses. iTBS produces sustained enhancement, whereas cTBS is associated with lasting suppression of cortical activity, indexed by potentiation and suppression of motor-evoked potential (MEP) following single-pulse TMS in the contralateral thumb muscle, respectively (43). It is believed that such lasting changes induced by iTBS and cTBS reflect LTP- and LTD-like mechanisms in the brain (43), and in previous experiments, they have been found to be mediated by NMDA receptor (44) and GABA receptor pathways (45), respectively.

### Paired associative stimulation

Paired associative stimulation is another well-established rTMS paradigm that has been associated with the induction of LTP-like neuroplasticity (PAS-LTP). It has been shown that PAS-LTP is mediated by NMDA receptors (46). The PAS protocol involves the repetitive delivery of two paired (180 pairs at 0.1 Hz for 30 min) stimulations: (1) an electrical peripheral nerve stimulation of the right median nerve, and 25 ms later, a (2) TMS pulse delivered to the contralateral motor cortex (M1) (hence PAS-25). PAS-25 results in LTP-like neuroplasticity that manifests as the potentiation of MEP in the thumb muscle following single-pulse TMS (46).

### Safety of rTMS in ASD

Available limited data indicate that rTMS, when applied within established safety guidelines, is well tolerated and safe in both adult and pediatric ASD populations (47, 48). There is no current evidence of increased risk of seizure (48).

### rTMS studies assessing neuroplasticity in ASD

Asperger’s disorder (AD), which was a subtype of the DSM-IV Pervasive Developmental Disorder, has now been subsumed under ASD in DSM-5 (1). A more direct evidence of aberrant neuroplasticity in AD subjects has been shown by recent rTMS studies using TBS and PAS paradigms. All these studies, however, have assessed neuroplasticity in the motor cortex (M1). One group found greater and long-lasting modulation of neuroplasticity (reflective of aberrant hyperplasticity) following both forms of TBS (cTBS and iTBS) in a small cohort (40) and, subsequently, in a relatively bigger sample of adults with AD (17). Another group, examining LTP-like neuroplasticity in a mixed cohort of adolescents and adults with AD using PAS, obtained similar results, i.e., aberrant neuroplasticity (18); however, the direction of aberrant neuroplasticity was different. In this study, it was found that, compared to typically developing subjects, PAS-induced LTP-like plasticity was significantly deficient (reflective of aberrant hypoplasticity) in the AD group.

## Assessing Neuroplasticity in ASD Subjects Using rTMS: Future Considerations

At present, research assessing neuroplasticity using rTMS in ASD population is at an early stage. Studies so far have only tested high-functioning ASD subjects at the motor cortex (M1). Furthermore, findings obtained in the adult population may not be generalized to the pediatric population. For example, Oberman et al. (47) found a “paradoxical facilitatory effect” to cTBS in more than one-third of their sample consisting of children and adolescents. Therefore, to what extent current findings can be generalized is certainly not very clear at present. The potential factors that need to be considered by future research are heterogeneity in the ASD population, potential impact of the presence/absence of comorbidities including intellectual disabilities, medication use, developmental age, site of stimulation, stimulation parameters (e.g., TBS versus PAS), etc.

The other important point for consideration is that all existing studies utilizing rTMS have assessed neuroplasticity at the motor cortex (M1) of ASD brain. In the future, studies need to look at neuroplasticity in other potential areas of interest in the ASD brain. Information regarding which sites to choose for assessing neuroplasticity in ASD brain may come from existing rTMS intervention studies. So far, studies that used rTMS for therapeutic purposes to improve either symptoms or physiological and cognitive indices have focused on four areas of ASD brain – the dorsolateral prefrontal cortex (DLPFC), medial prefrontal cortex (mPFC), supplementary motor area, and right pars triangularis and pars opercularis [for a review see Oberman et al. (49)]. The DLPFC was chosen due to its extensive network connection with other specialized distributed and local networks in brain (34). Dorsomedial PFC (dmPFC) is another key area for stimulation since it is believed to be uniquely linked with the mentalizing ability (50). A recent trial of deep rTMS delivered bilaterally to the dmPFC significantly improved social relatedness in ASD subjects (51). Therefore, both DLPFC and mPFC could be potential sites of interest for studying neuroplasticity in ASD. Other brain areas related to mentalizing, such as the temporoparietal junction (TPJ) (52), and facial processing, such as superior temporal sulcus (53), could be potential sites for stimulation as well.

Establishing a stimulation paradigm to reliably assess neuroplasticity from these key areas of brain is challenging; however, the combination of TMS with electroencephalography (TMS–EEG) offers researchers an exciting opportunity to gather a more direct measure of neuroplasticity from these areas of brain. Previously, our group established that TMS–EEG can be a reliable method to measure neuroplasticity from M1 and also DLPFC (54). More recently, using a pioneering technique that combines PAS with EEG – “PAS–EEG,” our group assessed and successfully demonstrated PAS-induced potentiation of cortical evoked activity, which is reflective of LTP-like neuroplasticity, in DLPFC (55). A similar TMS–EEG approach may be useful for studying neuroplasticity in other key areas of brain. For example, TBS can be combined with EEG to investigate neuroplasticity measures.

In the future, TMS–EEG can also be combined with various social–cognitive tasks and functional neuroimaging to better elucidate the brain–behavior relationship in ASD. Ultimately, TMS–EEG will be combined with genetic research to better elucidate the link between underlying genetic factors (i.e., polymorphisms) and aberration in neuroplasticity captured more directly by TMS–EEG cortical readout. Results from a few early exploratory studies assessing the impact of single-nucleotide polymorphisms, e.g., brain-derived neurotrophic factor valine-to-methionine substitution at codon 66 (Val66Met) genotype (56), on TMS-induced plasticity measures have so far been encouraging.

## Can rTMS be Used as a Therapeutic Tool to Rectify Aberrant Neuroplasticity in ASD?

Repetitive TMS affords researchers to design specific stimulation protocols that can modulate neuroplasticity, and such neuroplasticity-based brain stimulation interventions look promising. Recently, in a randomized double-blind sham-controlled study, our group demonstrated that application of 1,500 pulses/session of high-frequency (20 Hz) rTMS to DLPFC can “normalize” working memory deficits in schizophrenia (57). One possible mechanism of such improvement is enhancement of neuroplasticity in the DLPFC. There is a need to explore similar approach to treat aberrant neuroplasticity in ASD.

### What rTMS stimulation protocol to choose for stabilizing aberrant neuroplasticity in ASD?

Since aberrant neuroplasticity has been linked with the pathogenesis of ASD (7, 8), there is an urgent need to explore treatment paradigms that can stabilize aberrant neuroplasticity and thus potentially facilitate optimal social and cognitive performance and improve restricted and repetitive behaviors in ASD. In this regard, we would like to propose the potential role of extended dosing (i.e., 6,000 pulses) of high-frequency (i.e., 20 Hz) rTMS (58).

In healthy adults, rTMS applied on M1 has been shown to enhance GABA-mediated inhibitory neurotransmission indexed by lengthening of the cortical silent period (CSP), a CI measure reflective of GABA_B_-mediated inhibitory neurotransmission, with increased stimulation frequency. Our group found that the enhancement was maximal at 20 Hz (31). This finding breaks with convention that high-frequency stimulation results in excitation, whereas low-frequency stimulation results in inhibition, as 20-Hz rTMS, but not 1-Hz rTMS, resulted in a CSP prolongation (31, 58). One explanation is that 20-Hz rTMS may exert its inhibitory effect by selectively affecting networks involving fast-spiking inhibitory interneurons that mainly oscillate at higher (i.e., 30–70 Hz) frequencies (58). A recent study by our group investigating differing durations or doses of rTMS on CI in M1 in healthy subjects found that even a single session of extended dosing (6,000 pulses) with high-frequency (20 Hz) pulses led to significant lengthening of the GABA_B_-mediated CSP compared with other paradigms (58). This effect was not seen with active or sham 1- or 20-Hz rTMS at 1,200 pulses or 3,600 pulses.

It has been suggested that, depending on the direction and magnitude of inhibition, GABA_B_ receptor-mediated neurotransmission may attenuate neuroplasticity. In fact, baclofen, a GABA_B_ agonist, significantly attenuated LTP-like neuroplasticity in M1 induced by PAS (59). Since extended dosing (i.e., 6,000 pulses) of such specific high-frequency (20 Hz) rTMS protocol (58) appears to maximally enhance GABA_B_-mediated inhibitory neurotransmission, one approach would be to assess if such protocols are able to stabilize aberrant hyperplasticity seen in ASD. This line of approach is also consistent with the excitation–inhibition imbalance in ASD, i.e., a general deficit in GABA-ergic inhibition, an increased excitation/inhibition ratio (3), and an evidence of reduced expression of GABA_B_ receptors (33). In the future, proof-of-principle studies are needed to test this assumption. Because of its simplicity and reliability, such experiments may begin at M1 to see if the delivery of 6,000 pulses at 20 Hz can stabilize aberrant neuroplasticity in ASD subjects. If successful, further pilot studies will be required to assess whether rectifying aberrant neuroplasticity translates into actual clinical improvement or not. These pilot studies may potentially stimulate key areas of ASD brain discussed above, i.e., DLPFC, TPJ, and dmPFC, and determine key stimulation parameters, duration of sessions, etc.

## Conclusion

In summary, existing genetic and animal studies of ASD and evidence emerging from human rTMS studies have consistently indicated aberrant neuroplasticity in ASD brain. However, at this point, there are many unanswered questions regarding the exact etiopathological connection between aberrant neuroplasticity in the brain and development of autistic symptoms. Nevertheless, existing evidence still indicates that aberrant neuroplasticity could play a critical role in the pathogenesis of ASD. Therefore, it can be postulated that it may be possible to attain optimal social and cognitive performance in ASD by stabilizing aberrant neuroplasticity. In this context, we discussed a novel mechanism-driven approach toward achieving such goal using rTMS. If successful, this information will not only help us better understand the brain mechanisms involved in ASD but also stimulate trials testing mechanism-driven novel brain stimulation treatment paradigms for ASD.

## Conflict of Interest Statement

The authors declare that the research was conducted in the absence of any commercial or financial relationships that could be construed as a potential conflict of interest.
